# Dynamics and determinants of human plasma bile acid profiles during dietary challenges

**DOI:** 10.3389/fnut.2022.932937

**Published:** 2022-07-28

**Authors:** Jarlei Fiamoncini, Manuela J. Rist, Lara Frommherz, Pieter Giesbertz, Birgit Pfrang, Werner Kremer, Fritz Huber, Gabi Kastenmüller, Thomas Skurk, Hans Hauner, Karsten Suhre, Hannelore Daniel, Sabine E. Kulling

**Affiliations:** ^1^Department of Food Science and Experimental Nutrition, School of Pharmaceutical Sciences, Food Research Center – FoRC, University of São Paulo, São Paulo, Brazil; ^2^Department of Physiology and Biochemistry of Nutrition, Max Rubner-Institut, Federal Research Institute of Nutrition and Food, Karlsruhe, Germany; ^3^Department of Safety and Quality of Fruit and Vegetables, Max Rubner-Institut, Federal Research Institute of Nutrition and Food, Karlsruhe, Germany; ^4^Department of Nutritional Physiology, Technische Universität München, Freising-Weihenstephan, Germany; ^5^Biophysics I, Regensburg Center for Biochemistry, Universität Regensburg, Regensburg, Germany; ^6^Institute of Computational Biology, Helmholtz Zentrum München, German Research Center for Environmental Health, Neuherberg, Germany; ^7^Chair of Nutritional Medicine, Else Kroener-Fresenius-Centre for Nutritional Medicine, TUM School of Life Sciences, Technical University of Munich, Freising, Germany; ^8^Bioinformatics Core, Research Department, Weill Cornell Medicine in Qatar, Doha, Qatar

**Keywords:** OGTT, fasting, bile salts, postprandial, OLTT

## Abstract

In recent years, bile acids (BA) have received great interest due to their pleiotropic biological activity and the presence of plasma membrane-bound and nuclear receptors. Moreover, BA in blood have been identified by metabolite screening approaches as biomarkers that are associated with various diseases and even with a human longevity phenotype. With the growing interest in the microbiota contribution to the health-disease trajectory, BA that undergo deconjugation and other modifications by bacteria in the large intestine have become a prime target as a microbiome diversity modifier. We here profiled BA by a quantitative and a semiquantitative approach in 15 healthy and phenotypically very similar young individuals for over a 36-h fasting period, an oral glucose tolerance test (OGTT), and an oral lipid tolerance test (OLTT). We demonstrate a remarkable heterogeneity of the responses and describe the different dynamics of the plasma changes that likely originate from different routes by which BA enters the peripheral blood, and that may represent a direct secretion from the liver into the blood and a route that reaches the blood as a spill-over after passing from the gallbladder through the intestine and the portal system. We discuss the finding that an individual transport process involved in the passage of BA could be a critical determinant in the kinetics of plasma appearance and the overall phenotypic variability found.

## Introduction

Bile acids (BA) are synthesized by the liver and stored in the gallbladder, where they make up the bile together with cholesterol and phospholipids. Food ingestion causes the release of a multitude of gastrointestinal hormones under which cholecystokinin (CCK) mediates the contraction of the gallbladder to release bile and bile acids (BA) into the duodenum. BA act as detergents and solubilize dietary lipids in micelles by drastically increasing the surface area of lipid droplets, allowing access and hydrolysis by pancreatic lipase. After emulsification, digestion, and absorption of lipids, BA are taken up by specialized transporters in apical and basolateral membranes and pass through the portal blood back to the liver, where they are taken up for reuse in the bile. Efficient absorption, mainly in the terminal ileum *via* secondary active transporters, ensures that only a small fraction of the BA is transferred into the colon and lost in feces. Here, BA are metabolized by the microbiota that carry out deconjugation, dehydroxylation, and oxidation reactions, thus converting primary BA into secondary BA, with different physical–chemical properties and bioactivities. BA can also influence the composition of this microbial community (1, 2). A recent review by Gregorio and Galantini (3) summarizes such important aspects of the chemistry and physiology of BA (3).

With the discovery of the Takeda G protein-coupled receptor (TGR5) and the nuclear Farnesoid X Receptor (FXR), a new role of BA emerged by their activity as ligands for acute and chronic regulation of cell function in the gastrointestinal tract, the liver, and various peripheral tissues. Through the activation of TGR5 and FXR, BA can regulate their own metabolism, inhibit gluconeogenesis, lipogenesis, inflammation, and stimulate fatty acid β-oxidation (4, 5).

Metabolomics studies have identified BA as the most responsive group of plasma metabolites in healthy human volunteers when challenged with an oral glucose tolerance test (OGTT) (6, 7). Although BA are efficiently extracted from the portal blood when passing the liver, a fraction of BA escapes this first-pass hepatic extraction and spills over to systemic circulation. This spillover is estimated to account for around 10% of cholic acid and 20–30% of chenodeoxycholic and deoxycholic acids (8). The increase in plasma BA concentrations in the postprandial period has been shown to depend on the extent of gallbladder contraction (9), which is ultimately influenced by the composition of the ingested meal (10, 11). However, there are other variables that influence the spillover of BA into the systemic circulation, such as the function and capacity of intestinal and hepatic BA transporters. We also demonstrated that sex plays a role in the plasma response of BA in the postprandial period (12, 13).

Considering that BA activate receptors in different cell types and modulate physiological responses and that Bas’ concentrations rapidly increase in systemic circulation after the intake of a meal, there is a need to better understand the regulation of these processes. This could provide insights into individual metabolic responses and associated disease risks. We here report the postprandial kinetics of plasma BA in 15 healthy male individuals that underwent three dietary challenges: an extended fasting period of 36 h, an oral glucose tolerance test (OGTT), and an oral lipid tolerance test (OLTT) under very controlled conditions. We present novel results on the dynamics and variability of the BA responses in plasma BA to catabolic and anabolic conditions.

## Materials and methods

### Study design and participants

The Human Metabolome (HuMet) study was performed at the Human Study Center of the Technical University of Munich in Freising, Germany and has been described in detail elsewhere (14). Briefly, 15 healthy men between 23 and 34 years old, classified as normal weight (BMI ranging from 20 to 25 kg/m^2^), were recruited to be as similar as possible based on phenotype and anthropometry. All volunteers remained in the study. Table 1 provides information about their anthropometric and basic metabolic markers.

**TABLE 1:** Anthropometric and metabolic markers of the study subjects (*N* = 15).

Parameter	Unit	Mean	SEM	Minimum	Maximum
Age	years	28.20	0.81	23	34
Height	m	1.83	0.01	1.71	1.92
Body weight	kg	77.53	1.83	63.5	90.4
BMI	kg/m^2^	23.07	0.46	20.4	25.5
Diastolic BP	mmHg	121.50	3.12	106	160
Systolic BP	mmHg	81.93	1.54	70	93
Body fat	% of BW	15.06	1.17	8.8	22.6
Body fat	kg	14.43	0.86	8.84	19.25
Cholesterol	mg/dL	169.80	9.68	124	253
Triglycerides	mg/dL	78.13	6.77	50	125

Data expressed as mean and standards error of the mean.

In two periods of 2 days each, subjects underwent six different challenges, namely 36 h of fasting (from now on referred to as extended fasting), an oral glucose tolerance test (OGTT), a standard liquid diet test (SLD), an oral lipid loading test (OLTT), a physical activity test, and a stress test. A total of 56 blood samples were collected from each individual. The present report only presents data from the extended fasting, OGTT, and OLTT periods.

For the OGTT, a commercial OGTT test solution with 75 g of glucose (Dextro O.G.T.; RocheDiagnostics, Mannheim, Germany) was used. The OLTT was performed with a meal consisting of three parts of Fresubin Energy Drink (fiber-free and chocolate flavor; Fresenius-Kabi, Bad Homburg vor der Höhe, Germany) and one part Calogen (Nutricia, Zoetermeer, Netherlands)—a fat emulsion containing 50 g of long-chain triglycerides per 100 ml. The volume of the meal was calculated to provide 35 g of fat/m^2^ of body surface area.

The extended fasting challenge was done on day 1 with the first sample collected at 08:00 AM, after a 12-h fasting and subsequent blood sampling every 2 h until 0:00 AM (28^th^ h of the fasting period) and the last blood sampling happening at 08:00 AM of the following day, completing the 36-h period. After refeeding and receiving a standard liquid diet (SLD), subjects were discharged from the study center, and a 4-week washout period was allowed before the continuation of the protocol. On the second visit to the study center, the OGTT was performed after 12h fasting at 8:00 AM. After the ingestion of the glucose solution, blood was collected at 15, 30, 45, 60, 120, 180, and 240 min. After this, an SLD was offered (Fresubin Energy Drink, fiber-free and chocolate flavor) at 12:00 PM and again at 07:00 PM, each containing one-third of the calculated required energy. At 08:00 AM of the following day, a fasting blood sample was collected followed by ingestion of the OLTT drink and blood collection at 30, 60, 90, 120, 180, 240, 300, 360, 420, and 480 min. On each visit to the study center, participants were provided with a standardized dinner.

In an additional study, six healthy young men were recruited to determine if chylomicrons entering the systemic circulation also transport BA into the blood. After collecting the fasting plasma samples, volunteers received the oral lipid load and were requested to eat or drink nothing except water until 2.5 h after the lipid load. Then, postprandial plasma was collected. All plasma samples were partitioned into aliquots suited for BA analysis or chylomicron preparation. All samples were frozen and stored at –20°C until analysis.

### Chylomicron preparation

Two milliliters of fasting plasma or 1 ml of postprandial plasma were transferred to centrifuge tubes (Beckmann, thin-wall, and ultra-clear, 13 × 51 mm), and 2 ml or 3 ml of 0.9% NaCl solution was carefully layered above the plasma. Samples were centrifuged in an ultracentrifuge (Beckmann Optima XL100-K) in a swingout 55 Ti rotor at 14,000 rpm at 10°C for 2 h. The white layer of chylomicrons was removed from the top using Pasteur pipettes and frozen at –20°C until analysis of BA. The remaining plasma/NaCl mixture was frozen and stored at –20°C until BA analysis.

### Bile acid analysis

Two distinct analytical platforms were employed for the analysis of BA: a targeted (quantitative) and an untargeted approach (semi-quantitative), described in detail below. All analyzed BA are presented in Table 2, together with information about their identification and detection (monoisotopic mass and MRM transitions), nomenclature, etc.

**TABLE 2:** Information about the bile acids analyzed.

Common name	Abbrev.	Class according to metabolism	Class according to conjugation	Exact mass (–1)	Ret. Index (semi-quant. method)	MRM transitions (quant. method)	Ret. time (quant. method)	KEGG
Glycochenodeoxycholate	GCDCA	Primary	Gly-conj.	448.3068	5236.1	448.2 – 73.9	3.64	C05466
Glycochenodeoxycholate glucuronide	GCDCA-gluc	Primary	Gly-conj.	311.6658	4806	–	–	–
Glycocholate	GCA	Primary	Gly-conj.	464.3018	5163	464.2 – 74.1	2.18	C01921
Glycodeoxycholate	GDCA	Secondary	Gly-conj.	450.3214	1302	448.2 – 73.9	3.99	C05464
Glycohyocholate	GHCA	Secondary	Gly-conj.	464.3018	5020	–	–	–
Glycolithocholate	GLCA	Secondary	Gly-conj.	432.3119	5357.1	432.3 – 73.9	5.53	C15557
Glycoursodeoxycholate	GUDCA	Secondary	Gly-conj.	448.3068	5033	448.2 – 74	2.11	–
Glycocholate glucuronide	GCA-gluc	Primary	Gly-conj. and glucurinated	640.3339	4533	–	–	–
Glycochenodeoxycholate sulfate	GCDCA-sulf	Primary	Gly-conj. and sulfated	263.6282	4820	–	–	–
Glycocholenate sulfate	GCE-sulf	Secondary	Gly-conj. and sulfated	254.6229	4750	–	–	–
Glycodeoxycholate sulfate	GDCA-sulf	Secondary	Gly-conj. and sulfated	263.6282	4875	–	–	–
Glycolithocholate sulfate	GLCA-sulf	Secondary	Gly-conj. and sulfated	255.6307	5011	–	–	C11301
Tauro-beta-muricholate	TBMCA	Primary	Tau-conj.	514.2844	4774	–	–	–
Taurochenodeoxycholate	TCDCA	Primary	Tau-conj.	498.2895	5250	498.1 – 79.7	3.83	C05465
Taurocholate	TCA	Primary	Tau-conj.	514.2844	5150	514.2 – 79.8	2.39	C05122
Taurodeoxycholate	TDCA	Secondary	Tau-conj.	498.2895	5257.4	498.2 – 79.9	4.15	C05463
Taurolithocholate	TLCA	Secondary	Tau-conj.	482.2946	5345	482.2 – 80.1	5.64	C02592
Tauroursodeoxycholate	TUDCA	Secondary	Tau-conj.	498.2895	5025	498.2 – 80.1	2.31	–
Taurocholenate sulfate	TCE-sulf	Secondary	Tau-conj. and sulfated	279.6142	4750	–	–	–
Taurolithocholate 3-sulfate	TLCA-3-sulf	Secondary	Tau-conj. and sulfated	280.6221	5026.7	–	–	C03642
3b-hydroxy-5-cholenoate	3-OH-5-CE	Secondary	Unconj.	373.2748	5205	–	–	–
3beta,7alpha-dihydroxy-5-cholestenoate	3,7-OH-5-CE	Secondary	Unconj.	431.3167	5275	–	–	C17335
Chenodeoxycholate	CDCA	Primary	Unconj.	391.2854	5264	391.1 – 391.1	7.56	C02528
Cholate	CA	Primary	Unconj.	407.2803	5165	407.2 – 407.2	5.01	C00695
Deoxycholate	DCA	Secondary	Unconj.	391.2854	5294	391.1 – 391.1	7.8	C04483
Hyocholate	HCA	Secondary	Unconj.	407.2803	5046	–	–	C17649
Lithocholate	LCA	Secondary	Unconj.	375.2907	–	375 – 375	11.25	C03990
Ursodeoxycholate	UDCA	Secondary	Unconj.	391.2854	5055	391.1 – 391.1	5.58	C07880

The table provides general information about each compound as well as data regarding their identification on the two analytical platforms. The presence of a value for the retention index indicates that the BA was analyzed on the semi-quantitative platform. The presence of an MRM transition indicates that the referred metabolite was analyzed through the quantitative platform.

#### Quantitative bile acid analysis

Chemicals were purchased and used as described before (15). In addition, glycolithocholic acid (GLCA) was purchased from Toronto Research (Toronto, Canada). Standard solutions and calibration curves were prepared as described previously (15, 16).

##### Sample preparation

Plasma samples were stored at -80°C until analysis and were allowed to thaw on ice. Aliquots of 20 μl plasma were acidified with 50 μl of 0.005% HCOOH. Acidification of the samples was chosen to improve the recovery of BA as they are bound to the serum albumin (16). The degree of acidification was tested in separate trials to avoid deconjugation of the conjugated BA (data not shown). Internal standards were added to the acidified samples.

Sample clean-up was done by protein precipitation using the Phenomenex Impact™ protein precipitation plates (2 ml). Five hundred microlitres of ice-cold MeOH/ACN (50:50, v:v) were dispensed into each well, and the prepared plasma samples were transferred directly into the solvent. The plate was placed on an automatic stirrer for 15 min (700 rpm, 4°C). Subsequently, a partial vacuum was applied to filter the sample into a 96-well collection plate (2 ml). The residues were dried completely in a vacuum concentrator at 45°C and reconstituted in 100 μl MeOH. Samples were centrifuged for 20 min (4,700 *g*, 4°C), and 10 μl of the supernatant was used for analysis.

##### High-performance liquid chromatography coupled to mass spectrometry method

For the quantitative determination of BA from the plasma samples, the chromatographic parameters as previously described (15) were transferred to an analytical system consisting of a Nexera LC system (Shimadzu Europa GmbH, Duisburg, Germany) coupled with a 5500 Q-Trap mass spectrometer (Sciex, Darmstadt, Germany). Electrospray ionization in the negative mode was performed for BA and their conjugates (1–12.8 min). Source parameters were as follows: 40 psi (curtain gas), 600°C (Source Temperature), –4,500 V (Ion Spray Voltage), and 50 psi/60 psi (Ion Gas 1 and 2, respectively). Data were recorded in the multiple reaction monitoring modes (MRM) with nitrogen as collision gas. System operation and data acquisition were done using the Analyst 1.5.2. software (AB Sciex). Prior to sample measurement, the method was validated according to the FDA guidelines for bioanalytical method validation (17).

Quality control (QC) samples were generated from pooled EDTA plasma samples of four volunteers. After centrifugation, the supernatant plasma was pipetted off and stored as 150 μl aliquots at –80°C.

The stability of samples in the autosampler during measurements and the stability of the method throughout the whole study were ensured by inserting the QC samples into the batch. The first, the last and every seventh sample of a batch consisted of such a QC sample. Throughout the whole measurement period (FAST + OLTT), the QC plasma was extracted 20 times and injected 92 times. CVs were determined for each analyte within one batch and within all measured batches. According to the FDA specifications, the precision of the measured concentrations in and between the batches of 15% (20% if close to LOD) was aimed for, provided that the concentration measured in the QC sample was above the limit of quantification.

For the quantitative determination of BA in the chylomicron fraction, in the respective remaining plasma/NaCl mixture, and the corresponding plasma samples, the LC-MS/MS stable isotope dilution assay as previously described was used, except for the addition of one bile acid (GLCA; see transitions in Table 2) (15).

#### Semi-quantitative bile acid analysis

The semi-quantitative analysis of BA (untargeted metabolomics) was performed by Metabolon (Durham, United States) as described by Suhre et al. (18).

##### Sample preparation

Samples were prepared using an automated system (MicroLab STAR^®^ Hamilton Company, Reno United States). Recovery standards were added prior to the first step in the extraction process for quality control purposes. Samples were deproteinized with methanol under vigorous shaking for 2 min, followed by centrifugation. One fraction of the resulting extract was dried and reconstituted in the solvent containing a series of standards to ensure chromatographic consistency. A pooled sample generated by a small volume of each sample served as a technical replicate, and extracted water samples served as process blanks, both injected throughout the dataset. A cocktail of quality control standards was spiked into every sample, allowing instrument monitoring and chromatographic alignment.

##### UPLC-MS method

The method used a Waters ACQUITY ultra-performance liquid chromatography (UPLC) and a Thermo Scientific Q-Exactive high-resolution mass spectrometer interfaced with a heated electrospray ionization (HESI-II) source operated in negative mode. The orbitrap mass analyzer was operated at 35,000 mass resolution. The extracts were gradient-eluted from a C18 column (Waters UPLC BEH C18-2.1 × 100 mm, 1.7 μm) using a gradient consisting of methanol and water with 6.5 mM of ammonium bicarbonate at pH 8. The MS analysis alternated between MS and data-dependent MS*^n^* scans using dynamic exclusion and the scan range covered 70–1000 *m*/*z*. The only exception was for glycodeoxycholic acid, which was measured in the positive mode after a chromatographic separation in the same column using a mobile phase consisting of methanol, acetonitrile, water, 0.05% perfluoropentanoic acid, and 0.01% formic acid, operated at an overall higher organic content.

Several types of controls were analyzed in concert with the experimental samples: a pool of well-characterized human plasma served as a technical replicate throughout the dataset; extracted water samples served as process blanks; and a cocktail of QC standards that were carefully chosen not to interfere with the measurement of endogenous compounds was spiked into every analyzed sample, allowed instrument performance monitoring and aided chromatographic alignment.

##### Data extraction, compound identification, quantification, and normalization

Raw data were extracted, peak-identified, and quality control process using Metabolon’s hardware and software. Compounds were identified by using the Metabolon library based on authenticated standards that contain the retention time/index (RI), mass to charge ratio (*m/z*), and chromatographic data (including MS/MS spectral data) of all molecules present. Furthermore, biochemical identifications are based on three criteria: retention index within a narrow RI window of the proposed identification, accurate mass match to the library +/-10 ppm, and the MS/MS forward and reverse scores between the experimental data and authentic standards.

Peaks were quantified using the area under the curve. Data normalization was performed to correct variation resulting from instrument inter-day tuning differences by registering the medians to equal one (1.00) and normalizing each data point proportionately.

### Lipoprotein profiling using nuclear magnetic resonance

Lipoproteins were analyzed by nuclear magnetic resonance (NMR) spectroscopy based on a patented technology (U.S. 7,927,878; Australia 2005250571; Germany 10 2004 026 903 B4). Briefly, diffusion-weighted NMR spectra of blood plasma were recorded on a Bruker Avance II plus 600-MHz spectrometer (Bruker BioSpin, Ettlingen, Germany), which revealed characteristic overall profiles of the lipoprotein signals. The spectral regions ranging from 1.5 to 0.7 ppm were modeled into a set of 15 lipoprotein subfractions. These 15 lipoprotein subfractions were used to calculate lipoprotein size and quantity (number) in terms of the concentration (nM) of particle subclasses and the average particle size (nm), of which only the chylomicrons are presented here.

### Data analysis/statistics

**FIGURE 1:**
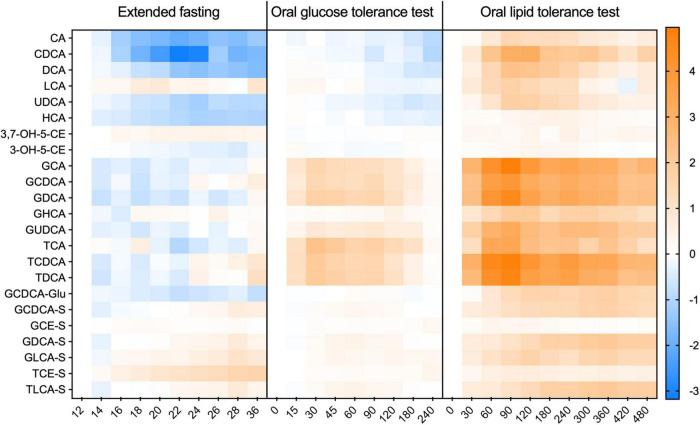
Kinetic profiles of individual bile acids in plasma during the extended fasting and dietary challenges. The appearance of individual BA species in each challenge expressed as a fold-change from the baseline. Data was Log-2 normalized and presented as mean ± SEM. *N* = 15 with the *X*-axis given in minutes.

Data from both analytical platforms are expressed as mean ± standard error of the mean. In the case where data from the untargeted (semi-quantitative BA analysis) is used, the intensity of the signal for each BA was normalized by the baseline in each dietary challenge, being expressed as a fold-change in relation to the baseline (12 h of fasting). Comparisons of BA values in time series (different time points in each dietary challenge) were made using a mixed-effects model and correcting for multiple comparisons using Tukey’s test. Differences with a *p*-value of < 0.05 were considered statistically significant. For the heatmap depicted in Figure 1, concentrations or intensities of BA were Log2 normalized and expressed as a fold-change from the baseline. GraphPad Prism version 9 was used for data analysis and for the preparation of the graphs.

## Results

### The overall bile acid response in plasma after different dietary challenges

Two different analytical platforms were employed to analyze the plasma BA profiles in response to the challenges. We mainly present the data obtained by quantitative analysis, which, however, did not include sulfated and glucuronidated BA. For those two BA classes, we present data only as the fold-change from the baseline. For the BA species measured in both platforms, the results were similar and comparable in terms of changes over time and relative proportions of individual entities. Supplementary Table 1 provides the concentration of each class of BA, only considering the BA analyzed by the quantitative platform.

The postprandial appearance of BA in plasma shows a remarkable inter-individual variability. Samples from the challenges described here with a focus on BA were also analyzed for ∼600 other plasma metabolites (14), but BA displayed an extreme variability exceeding that of most other metabolites profiled.

**FIGURE 2:**
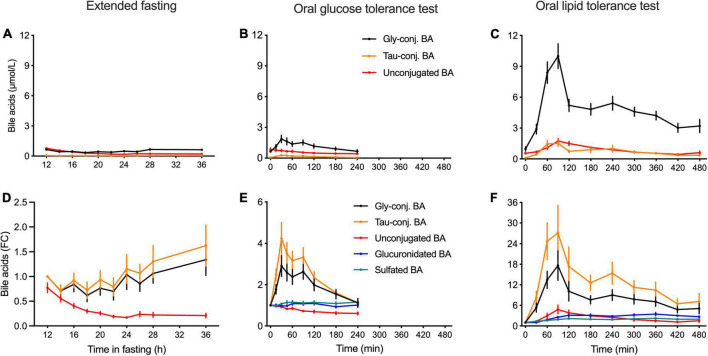
Kinetics of classes of bile acids in plasma in the postprandial state and during the extended fasting period. **(A–C)** Plasma concentration of BA displayed as the sums of unconjugated, glycine and taurine-conjugated BA is expressed as nmol/L. **(D–F)** Appearances of unconjugated, glycine- and taurine-conjugated BA as well as sulfated and glucuronidated BA species are expressed as the fold-change from the baseline. **(A,D)** Samples from the extended fasting period. **(B,E)** Samples from the OGTT. **(C,F)** Samples from the OLTT. Data presented as mean ± SEM (*N* = 15).

Both in the OGTT and OLTT, a very large postprandial increase in plasma concentrations of BA conjugated to glycine and taurine was observed in comparison to the baseline values obtained after 12 h of fasting (Figure 2). Plasma levels of unconjugated BA decreased by around 30% during the OGTT, while they increased almost 4.8-fold in the OLTT (Figures 2E,F).

In the OLTT, the challenge that elicits a near-maximal response in BA secretion caused taurine-conjugated BA to increase almost 27-fold at *t* = 90 min when compared to the baseline, while glycine-conjugated BA increased around 20-fold (Figure 2B). In the OGTT, taurine-conjugated BA increased on average 3.8-fold and glycine-conjugates 2.7-fold (Figure 2E). Plasma levels of unconjugated BA showed a 4.8-fold increase during the OLTT, reaching *C*_max_ between 1 and 2 h after intake of the test meal. During the OGTT, the concentrations of these BA decreased in comparison to fasting levels, with values around 40% lower at *t* = 180 min (Figure 2B) than at the test start. Plasma concentrations of glucuronidated and sulfated BA displayed a 3.1- and 2.1-fold increase during the OLTT, respectively, while remaining unchanged during the OGTT (Figure 2B).

In the extended fasting period, a strong decrease in the concentration of unconjugated BA was observed. These compounds decreased by 75% at 24 h of fasting as compared to the baseline (after 12 h of fasting). Plasma levels of conjugated BA remained unchanged throughout the fasting challenge. Plasma levels of GCDCA decreased by 40% after 22 h of fasting, while the concentration of the sulfated BA species displayed a steady increase reaching levels higher by 80% than those at the baseline (Figure 2B). The heatmap in Figure 1 shows the plasma kinetics of individual BA species expressed as a fold-change from the baseline (12-h fasting) in each challenge after the Log2-normalization of the concentrations.

### Changes in the composition of plasma bile acid profiles during different dietary challenges

**FIGURE 3:**
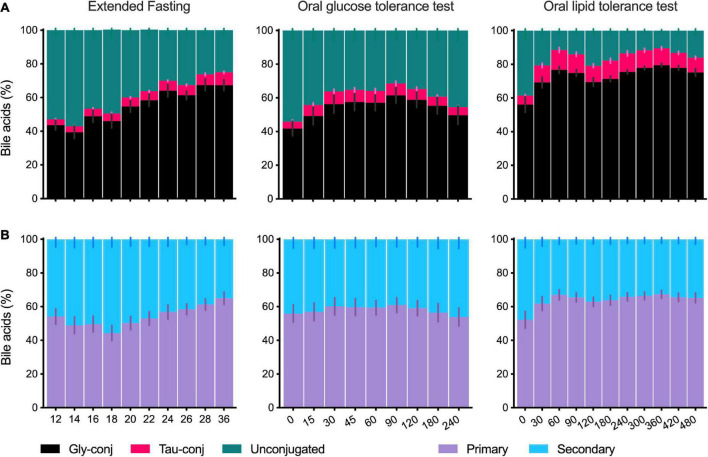
Composition of the plasma bile acid profile in the postprandial state and during the extended fasting. **(A)** Percentage of the sums of unconjugated, glycine- and taurine-conjugated BA in the total profile as collected during the extended fasting, OGTT and OLTT. **(B)** Percentage of the sums of primary and secondary BA in the plasma BA profile collected during the extended fasting, OGTT and OLTT. Data presented as mean ± SEM (*N* = 15). The *X*-axis for fasting is given in hours and the *X*-axis for OGTT and OLTT is in minutes.

After overnight fasting, the plasma BA profile comprises unconjugated and glycine-conjugated BA making up to 40–60%, while taurine-conjugated BA corresponds to 5–10% of the total plasma pool (Figure 3A). These proportions change when fasting is extended or the OGTT and OLTT challenges are performed. The OLTT induced the most impressive increase in glycine- and taurine-conjugated BA that went from 56 and 5% at fasting to 76 and 12%, respectively, at *t* = 60 min. The changes induced by the OGTT showed the same direction, although with a smaller magnitude. When fasting was extended to 36 h, the proportion of unconjugated BA decreased from 53 to 25% of the total BA pool.

When BA are classified according to synthesis into primary and secondary BA, there is a balanced proportion, each corresponding to 40–60% of the total plasma BA profile (Figure 3B), and this ratio is only modestly influenced by fasting or other challenges (Figure 3A). Noteworthy is the decrease in primary BA starting at 18 h of the extended fasting period (reaching 44%, in contrast to 54% at the baseline) and recovery in levels toward the 36 h period. Also, in the OLTT, a change in the ratio between primary and secondary BA was observed, with the participation of primary BA increasing from 52% (*t* = 0 min) to 67% already in the first hour (Figure 3B).

### Dynamics and variability of plasma changes

**FIGURE 4:**
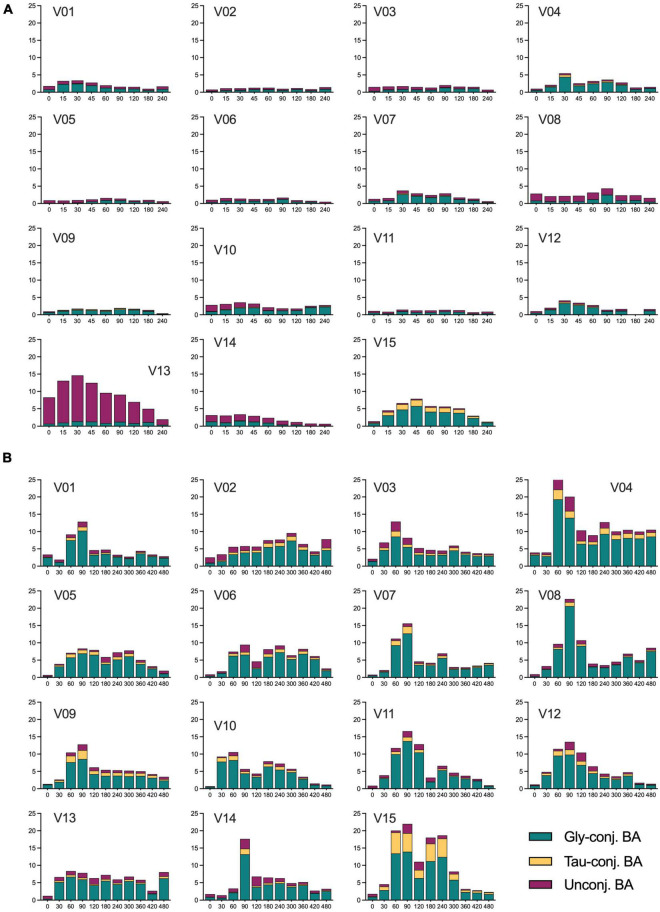
Concentration of unconjugated, glycine- and taurine-conjugated BA in plasma samples of all subjects during the OGTT and OLTT. **(A)** Concentrations of the three classes of BA in each individual during the OGTT. **(B)** Concentrations of the three classes of BA in every subject during the OLTT. V01, V02… V15 refer to individual study subjects. The concentration of the BA classes is expressed as μmol/L with the *X*-axis given in minutes.

Bile acids are amongst the endogenous metabolites with high-interindividual variability (13, 19, 20). This is true for the fasting and the postprandial states when these differences become even more evident. Figure 4 shows the range of BA plasma concentrations in each subject during the OGTT and the OLTT, illustrating the high interindividual variability. Despite the meticulous recruitment of the 15 individuals with very similar BMI, age, and overall health and fitness status, the variability of BA profiles is impressive and also different when the OGTT and the OLTT responses are considered separately. For instance, subject 13 has a distinct profile with very high concentrations of unconjugated BA already in the fasting state, which is further increased during the OGTT (Figure 4A). Subject 09, on the other hand, has only a small fraction of unconjugated species. Subject 15 displayed a very pronounced increase of glycine- and taurine-conjugated BA during the OGTT and reached the highest plasma concentration of taurine-conjugated BA of all (Figure 4A). Although the plasma levels of BA are much higher during the OLTT than during the OGTT, subject 15 is the individual with the highest increase of conjugated BA in circulation (Figure 4B).

To assess the intestinal absorption of BA as a critical variable, their appearance in plasma in the postprandial periods was compared to that of glucose and chylomicrons. Glucose absorption is very fast and occurs predominantly in the upper parts of the small intestine, whereas chylomicron appearance in blood takes much longer due to fat digestion, assembly of chylomicrons in the epithelial cells, and transfer *via* the lymph into the blood.

**FIGURE 5:**
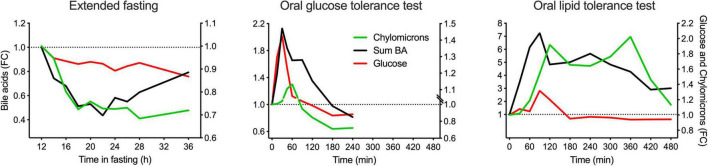
Comparison of the kinetic behaviors of BA (total), glucose, and chylomicrons in plasma in the postprandial state and during the extended fasting period. Plasma concentration of the sum of all BA quantified is represented in the left *Y*-axis, whereas plasma concentration of chylomicrons and glucose are represented in the right *Y*-axis. Data are expressed as the fold-change relative to the baseline and presented as mean ± SEM (*N* = 15).

In the OGTT, the *T*_max_ of BA (sum of all) was identical to the *T*_max_ for glucose. The OLTT BA profile revealed the first peak at 90 min and a second peak at 240 min. The *T*_max_ in the OLTT was again identical to that of glucose, which also had its *T*_max_ in the OLTT at 90 min. This means that BA absorption occurs as fast as that of glucose, with a delay of around 60 min for both glucose and BA because the high fat/high energy density of the OLTT delays gastric emptying markedly. In addition, glucose is absorbed in the duodenum and the jejunum; we need to conclude that this also applies to BA absorption in the OGTT and for the first BA peak in the OLTT plasma profile. It appears plausible that the second peak in the BA profile that also forms the larger shoulder represents the absorption of BA from more distal regions of the small intestine *via* the sodium-dependent BA transporter considered to be the prime pathway in the enterohepatic recycling of BA and responsible for minimizing the loss of BA to the colon. In the extended fasting period, the decline in chylomicrons matched the decline in the concentration of the total BA pool (Figure 5).

Since chylomicrons contain cholesterol and cholesterol esters, we assumed they might also contain some BA. Therefore, in an additional small study, chylomicrons were isolated after the OLTT, and the BA were profiled in the chylomicrons as described in the Methods section. Analysis revealed that only about 2–5% of all BA in plasma are associated with the chylomicron fraction (data not shown).

## Discussion

The appearance of the different BA in peripheral blood depends on the quantity and quality of food ingested and seems predominantly affected by the magnitude of gallbladder contraction (9). As previously described (11, 21), we demonstrate that the response after a fat-rich liquid test meal causes an increase in BA in peripheral blood that exceeds after an OGTT almost 8-fold (Figure 2). The BA profile in the peripheral blood is the result of many biological processes with distinct characteristics that all are concentration-dependent and have certain substrate specificities (12, 22). It starts with different output quantities upon stimulation of gallbladder contraction by CCK, followed by the uptake of BA into intestinal epithelial cells mediated by at least two (possibly more) other transport systems in apical membranes of epithelial cells with different substrate specificities, and similarly, discriminant transporters in the basolateral membrane the export BA into the portal system. Liver flow-through means that again depending on the concentration and pattern of BA presented to the transporters in the hepatocyte basolateral membranes, BA are taken up with different substrate specificities and kinetics. When the peripheral blood is analyzed, only a small fraction of these BA passing the liver will appear in the blood and their signature is likely not similar to the profile found in the bile and released from the gallbladder. In addition, for almost all transporters involved in the handling of the BA, genetic heterogeneity with consequences in kinetics and differences in expression level are known; the latter is even partly controlled by the BA pool itself *via* FXR and other transcription control mechanisms. We have recently shown in a different cohort that a single nucleotide polymorphism in the OATP1A2 (SLCO1A2) transporter—known to be functionally relevant—led to quite remarkable differences in the postprandial BA concentration profiles in an OGTT in comparison to non-carriers (13). Since OATP1A2 has also been identified in cholangiocytes of the liver (23), the genetic variant could as well cause a change in the composition of the bile even before it reaches the bladder for further concentrating processes. Given the fact that dozens of proteins are participating in the enterohepatic cycling of BA, and all can have functionally relevant structural differences and different expression levels, the heterogeneity in BA profiles observed here in the 15 otherwise very homogenous volunteers (14) reveals a unique feature of these metabolites that also carry the biological activity. Different BA have different binding affinities for TGR5 and FXR, which means that their biological effects may vary considerably between individuals. Even when BA are identified as biomarkers associated with distinct disease risks in cohort studies or even as markers of overall lifespan in humans (24), the translation into the biological activity that they may elicit has likely also a huge interindividual variability (19).

A remarkable observation is the higher levels (and proportion) of unconjugated BA during the OGTT than during the OLTT (Figure 3). The pattern of the BA during the OGTT shows similarities to those observed in the fasting state, with unconjugated species making up around 50% of the total pool. As shown in Figure 4, in some individuals, these species are found in relatively large concentrations in the OGTT, whereas the same individuals show a different BA pattern in the OLTT. Although it cannot be proven here, it seems biologically plausible that the blood profiles of BA in the fasting state and the OGTT have a different origin than those found in the OLTT. In the fasting state and during the OGTT, the BA in peripheral blood may originate directly from an efflux from hepatocytes and may not have their origin exclusively in influx from the intestine. That was already postulated some time ago as a cause of the several-fold increased BA levels during the OGTT (7). In support of this postulate, sulfated or glucuronidated species were in essence not detectable (or below detection level) in plasma during the OGTT, which may mean that they are not released from the liver into the blood directly. The prime export system in the canalicular hepatocyte membrane is the BSEP protein with some ATP-dependent transporters, such as MRP2, also contributing to BA flux into bile (25), while the basolateral membrane of hepatocytes contains two main BA transport systems. One (NTCP) is a true importer that mediates influx from the blood into the cell in a sodium-dependent manner; the other is a protein (OATP1B1) that can operate in an exchange mode. Since Ostα/β is found in this membrane domain together with some ATP-dependent export pumps of the MRP-series (26), various hepatic transport proteins are capable of exporting BA directly from hepatocytes into the blood. This pathway may represent an important route by which BA reach peripheral blood in the fasting state but even during the OGTT with a substantial efflux of BA (mainly unconjugated species) from the liver into the blood. There is ample evidence from animal studies that insulin and blood glucose level have significant effects on hepatic bile handling and on the bile-salt-independent fraction of canalicular bile formation (27, 28). In the OGTT, the blood BA pool may represent a hepatic secretion fraction, but also a significant portion of BA that comes from the intestine *via* the liver and contains glycine- and taurine-conjugated species—although in a lower proportion than in the OLTT.

What is also an obvious difference in the BA profiles during the OGTT and the OLTT are the time points when the *C*_max_ values are reached. In the case of the OGTT, the peak of the BA (sum of all species) can be superimposed onto the glucose peak in plasma. That suggests that the BA are absorbed in the most proximal parts of the small intestine. Here, the OATP1A2 transporter acts as a uniporter, allowing BA to be taken up by enterocytes *via* the lumen to a cell concentration gradient. As shown by Sonne et al. (9), the OGTT causes an ejection volume of the gallbladder of around 20%, and that can be increased to >60% when a high-fat liquid meal is provided to the same volunteers (9). That means that in the OGTT the BA – that peak at 30 to 60 min in blood – are in part of bladder origin with absorption *via* the OATP1A2 transporter in the duodenum/jejunum. In the OLTT, however, not only the bladder ejection volume increases with a much higher BA concentration in the intestinal lumen, but also the time when *C*_max_ is reached for glucose and for BA. With paracetamol as a marker, Sonne et al. (9) demonstrated a markedly reduced appearance rate of paracetamol in plasma when administered together with high fat, likely by the high energy density delaying gastric emptying. In the present study, we also observed for glucose and BA that the *C*_max_ was delayed by almost 60 min. Yet, the initial BA peak could be superimposed onto the glucose peak, suggesting that this initial BA fraction is also absorbed like glucose in the upper small intestine—mainly *via* OATP1A2. As a driving force for this fast absorption in the upper small intestine, the high-luminal BA concentration could exceed 25 mM in the lumen (29). The majority of the BA ejected from the gallbladder, however, may be trapped in mixed micelles formed from the lipid droplets in the OLTT, and thus may follow fat digestion and absorption of free or glycerol-bound fatty acids and move further down toward the terminal ileum. The apical import of BA in the ileum is mediated by the sodium-dependent ASBT transporter that can transport BA even against the concentration gradient, allowing very efficient clearance from the small intestine and minimizing BA flux across the ileocecal valve. Exit from enterocytes into the portal system is mediated by the OST*a*/β transporters (30). Since we could also classify the chylomicrons appearing in blood *via* NMR, their *C*_max_ was further delayed by around 30 min (Figure 5) compared to glucose or the first BA peak. That may represent the time needed for assembling the particles in the intestinal epithelium and the transfer *via* the lymph before entering the blood. BA and chylomicron profiles are bimodal with a small second peak and a broad shoulder with elevated levels over the entire test period of 480 min. A second peak may well represent additional secretion of BA into the gut followed by reabsorption. Inspection of the individual BA profiles (Figure 4) reveals that not all subjects show a bimodal plasma profile suggesting that this phenomenon also represents an interindividual response mechanism.

## Conclusion

Taken together, we here demonstrate in a group of healthy young volunteers with very similar phenotypes that BA profiles in plasma show a remarkable intra- and interindividual variability. Both the fraction of unconjugated and the glycine- and taurine-conjugated BA species vary in proportion and absolute plasma levels across the extended fasting period and the two dietary challenges. It is proposed that the heterogeneity of the profiles originates from the secretion of BA (mainly conjugated) from the liver across the basolateral hepatocyte membrane by various transporters present in this membrane domain, and this process could dominate the fasting signature and be part of the OGTT response. In the OGTT and in the initial phase of the OLTT response, BA appear to be absorbed independent of fat digestion from the upper small intestine mainly *via* a uniporter system, while in the OLTT, an additional fraction of the BA—dominated by glycine- and taurine-conjugated species—is moved down to the ileum entrapped in the mixed micelles with reabsorption *via* a sodium-dependent transporter in the ileum. Genetic heterogeneity and differences in expression levels of dozens of proteins involved in BA handling and enterohepatic circulation may add to interindividual response signatures.

## Data availability statement

The original contributions presented in this study are included in the article/Supplementary material, further inquiries can be directed to the corresponding author.

## Ethics statement

The studies involving human participants were reviewed and approved by Technische Universität München (#2087/08). The patients/participants provided their written informed consent to participate in this study.

## Author contributions

HD, HH, and TS designed the study. MR, BP, LF, WK, and FH participated in the execution of the study and/or analysis. JF and PG analyzed the data. HD, GK, KS, and SK contributed with reagents, analytical tools, and expertise. PG, JF, MR, LF, and HD wrote the manuscript. All authors contributed to the article and approved the submitted version.

## Supplementary material

Supplementary Table 1 Concentrations of bile acids in plasma during three metabolic challenges. Data presented as mean and standard error of the mean. Concentrations are expressed in μmol/L. Time intervals during the extended fasting challenge are given in hours and during the OGTT and OLTT in minutes.
