# Supplementary material for: Dynamics and determinants of human plasma bile acid profiles during dietary challenges
Source: Front Nutr. 2022 Jul 28;9:932937. doi: 10.3389/fnut.2022.932937 (PMC9366195; doi:10.3389/fnut.2022.932937)
Supplement: Supplementary Table 1 — Concentrations of bile acids in plasma during three metabolic challenges. Data presented as mean and standard error of the mean. Concentrations are expressed in μmol/L. Time intervals during the extended fasting challenge are given in hours and during the OGTT and OLTT in minutes. [file Data_Sheet_1.pdf]

Time	Total			Primary			Secondary			Gly-conj			Tau-conj			Unconjugated		
	Mean	SEM	N	Mean	SEM	N	Mean	SEM	N	Mean	SEM	N	Mean	SEM	N	Mean	SEM	N
**Extended fasting**																		
12	1.47	0.19	15	0.78	0.13	15	0.69	0.11	15	0.64	0.10	15	0.05	0.01	15	0.77	0.10	15
14	1.02	0.12	15	0.50	0.08	15	0.52	0.08	15	0.43	0.08	15	0.04	0.01	15	0.55	0.07	15
16	0.90	0.11	14	0.44	0.08	14	0.46	0.07	14	0.45	0.08	14	0.04	0.01	14	0.41	0.05	14
18	0.69	0.13	13	0.31	0.08	13	0.38	0.07	13	0.35	0.10	13	0.04	0.01	12	0.30	0.04	13
20	0.73	0.12	15	0.37	0.06	15	0.36	0.07	15	0.44	0.09	15	0.04	0.01	15	0.26	0.04	15
22	0.61	0.11	15	0.33	0.07	15	0.28	0.05	15	0.38	0.09	15	0.03	0.01	14	0.19	0.03	15
24	0.70	0.10	15	0.42	0.08	15	0.27	0.04	15	0.48	0.09	15	0.04	0.01	15	0.17	0.02	15
26	0.71	0.11	15	0.43	0.09	15	0.29	0.04	15	0.43	0.06	15	0.04	0.01	15	0.24	0.05	15
28	0.93	0.20	15	0.58	0.15	15	0.35	0.07	15	0.65	0.15	15	0.06	0.01	15	0.22	0.05	15
36	0.91	0.14	15	0.60	0.11	15	0.31	0.05	15	0.63	0.11	15	0.07	0.01	15	0.21	0.05	15
**Oral lipid tolerance test**																		
0	1.61	0.26	15	0.88	0.18	15	0.73	0.12	15	0.97	0.22	15	0.09	0.02	15	0.55	0.09	15
30	4.01	0.56	15	2.62	0.47	15	1.39	0.16	15	2.90	0.49	15	0.43	0.09	15	0.68	0.11	15
60	10.94	1.41	15	7.57	1.08	15	3.36	0.45	15	8.39	1.04	15	1.45	0.37	15	1.10	0.20	15
90	13.26	1.47	15	8.77	1.01	15	4.49	0.61	15	9.99	1.20	15	1.53	0.31	15	1.74	0.25	15
120	7.43	0.74	15	4.68	0.52	15	2.75	0.32	15	5.20	0.60	15	0.74	0.14	15	1.49	0.21	15
180	6.85	0.91	15	4.46	0.72	15	2.38	0.28	15	4.83	0.57	15	0.90	0.31	15	1.12	0.11	15
240	7.29	1.02	15	4.81	0.73	15	2.48	0.36	15	5.42	0.67	15	0.99	0.33	15	0.89	0.08	15
300	5.92	0.57	15	3.87	0.35	15	2.04	0.28	15	4.59	0.44	15	0.67	0.12	15	0.65	0.05	15
360	5.29	0.53	15	3.48	0.29	15	1.81	0.27	15	4.21	0.42	15	0.54	0.08	15	0.54	0.05	15
420	3.82	0.54	15	2.49	0.34	15	1.33	0.23	15	3.02	0.45	15	0.36	0.07	15	0.44	0.05	15
480	4.16	0.78	15	2.65	0.46	15	1.51	0.35	15	3.20	0.64	15	0.36	0.08	15	0.60	0.15	15
**Oral glucose tolerance test**																		
0	2.00	0.49	15	1.17	0.33	15	0.83	0.19	15	0.65	0.07	15	0.06	0.01	15	0.84	0.16	14
15	2.81	0.78	15	1.71	0.51	15	1.10	0.29	15	1.13	0.20	15	0.16	0.06	15	0.77	0.14	14
30	3.71	0.89	15	2.42	0.63	15	1.28	0.29	15	1.86	0.36	15	0.27	0.10	15	0.74	0.13	14
45	3.23	0.79	15	2.07	0.56	15	1.16	0.26	15	1.63	0.34	15	0.25	0.11	15	0.66	0.12	14
60	2.77	0.57	15	1.70	0.40	15	1.07	0.21	15	1.38	0.24	15	0.20	0.08	15	0.66	0.12	14
90	2.76	0.56	15	1.75	0.39	15	1.01	0.20	15	1.52	0.25	15	0.20	0.08	15	0.56	0.11	14
120	2.18	0.43	15	1.36	0.30	15	0.82	0.16	15	1.16	0.22	15	0.15	0.06	15	0.49	0.08	14
180	1.69	0.32	14	1.03	0.23	14	0.66	0.12	14	0.91	0.17	14	0.09	0.03	14	0.45	0.08	13
240	1.23	0.17	15	0.71	0.14	15	0.52	0.07	15	0.67	0.15	15	0.06	0.02	15	0.42	0.07	14
